# Complement Overactivation and Consumption Predicts In-Hospital Mortality in SARS-CoV-2 Infection

**DOI:** 10.3389/fimmu.2021.663187

**Published:** 2021-03-25

**Authors:** György Sinkovits, Blanka Mező, Marienn Réti, Veronika Müller, Zsolt Iványi, János Gál, László Gopcsa, Péter Reményi, Beáta Szathmáry, Botond Lakatos, János Szlávik, Ilona Bobek, Zita Z. Prohászka, Zsolt Förhécz, Dorottya Csuka, Lisa Hurler, Erika Kajdácsi, László Cervenak, Petra Kiszel, Tamás Masszi, István Vályi-Nagy, Zoltán Prohászka

**Affiliations:** ^1^ Department of Internal Medicine and Haematology, Semmelweis University, Budapest, Hungary; ^2^ Research Group for Immunology and Haematology, Semmelweis University–Eötvös Loránd Research Network (Office for Supported Research Groups), Budapest, Hungary; ^3^ Department of Haematology and Stem Cell Transplantation, Central Hospital of Southern Pest National Institute of Haematology and Infectious Diseases, Budapest, Hungary; ^4^ Department of Pulmonology, Semmelweis University, Budapest, Hungary; ^5^ Department of Anaesthesiology and Intensive Therapy, Semmelweis University, Budapest, Hungary; ^6^ Department of Infectology, Central Hospital of Southern Pest National Institute of Haematology and Infectious Diseases, Budapest, Hungary; ^7^ Department of Anaesthesiology and Intensive Therapy, Central Hospital of Southern Pest National Institute of Haematology and Infectious Diseases, Budapest, Hungary

**Keywords:** SARS-CoV-2 infection, mortality, severity, complement system, coronavirus disease (COVID-19), complement activation and consumption

## Abstract

**Objectives:**

Uncontrolled thromboinflammation plays an important role in the pathogenesis of coronavirus disease (COVID-19) caused by SARS-CoV-2 virus. Complement was implicated as key contributor to this process, therefore we hypothesized that markers of the complement profile, indicative for the activation state of the system, may be related to the severity and mortality of COVID-19.

**Methods:**

In this prospective cohort study samples of 102 hospitalized and 26 outpatients with PCR-confirmed COVID-19 were analyzed. Primary outcome was in-hospital, COVID-19 related mortality, and secondary outcome was COVID-19 severity as assessed by the WHO ordinal scale. Complement activity of alternative and classical pathways, its factors, regulators, and activation products were measured by hemolytic titration, turbidimetry, or enzyme-immunoassays. Clinical covariates and markers of inflammation were extracted from hospital records.

**Results:**

Increased complement activation was characteristic for hospitalized COVID-19 patients. Complement activation was significantly associated with markers of inflammation, such as interleukin-6, C-reactive protein, and ferritin. Twenty-five patients died during hospital stay due to COVID-19 related illness. Patients with uncontrolled complement activation leading to consumption of C3 and decrease of complement activity were more likely to die, than those who had complement activation without consumption. Cox models identified anaphylatoxin C3a, and C3 overactivation and consumption (ratio of C3a/C3) as predictors of in-hospital mortality [HR of 3.63 (1.55–8.45, 95% CI) and 6.1 (2.1–17.8), respectively].

**Conclusion:**

Increased complement activation is associated with advanced disease severity of COVID-19. Patients with SARS-CoV-2 infection are more likely to die when the disease is accompanied by overactivation and consumption of C3. These results may provide observational evidence and further support to studies on complement inhibitory drugs for the treatment of COVID-19.

## Introduction

Three highly pathogenic coronaviruses for human populations appeared in the past two decades. First, severe acute respiratory syndrome (SARS) was described in 2003 when the emergence of a human pathogen coronavirus (SARS-CoV) was noted (1–3). Second, in September 2012, Middle East Respiratory Syndrome Coronavirus (MERS-CoV) was reported to cause human infection (4). Third, in December, 2019, a novel coronavirus (SARS-CoV-2) causing coronavirus disease (COVID-19) was initially identified in Wuhan, China, and caused later a pandemic starting in early March, 2020 (5). The rapid, global spread of the virus is presently ongoing, by late January, 2021, over 4.0 million cases per week are being reported worldwide, with around 70.000 deaths per week (6). Organ dysfunction, particularly complex coagulopathy and respiratory failure often in the context of acute respiratory distress syndrome (ARDS), and multiple organ failure are associated with highest mortality, especially in the elderly (7).

The complement system is generally considered as part of the first line defense against pathogens, including viral infections, and this fact is marked by the multiple mechanisms by which viruses avoid complement attack (8). On the other hand, complement (with or without the contribution of antibodies) may also play an enhancing role in viral infections, as described for HIV (9–11). Furthermore, complement activation, especially generation of anaphylatoxin C5a, is involved in the development of acute lung injury caused by influenza A (12). Animal experiments also supported the involvement of complement activation in the development of SARS caused by CoV infection (13). Accordingly, complement inhibitors are considered as promising therapeutic options during the ongoing SARS-CoV-2 pandemic (14, 15), and the first COVID-19 cases treated by the C5 inhibitor monoclonal antibody eculizumab (16–18), and by the C3-targeting small peptide inhibitor AMY-101 (19) have recently been reported. Further in line with these considerations, Annane et al. reported promising survival results in a proof-of-concept, non-randomized study of 80 eculizumab treated intensive care unit patients with COVID-19 (20).

Complement system is a sensitive enzymatic cascade responsive through its lectin pathway to carbohydrate patterns of pathogens, or through its classical pathway to antibodies (21, 22). The alternative pathway (AP) represents an amplification loop on the level of the central component C3, with generation of powerful biological mediators including anaphylatoxins, opsonins, and lytic complexes (23). Patients with various infections may benefit from laboratory evaluation of the complex system, which can show the presence of complement deficiency, activation state, or consumption of the whole system, or its dedicated pathways (24, 25). Two recent studies have shown a clear association between respiratory failure, COVID-19 outcome, and systemic complement activation (26, 27) in COVID-19 patients. Furthermore, a large-scale multi-omic analysis of COVID-19 severity mapped several molecular features including the complement system, and identified strong associations between complement activation and severe COVID-19 phenotype (28). In a recent comprehensive review Perico and colleagues described multiple mechanisms of how endothelial cells, and complement system particularly, may contribute to severe thromboinflammation in COVID-19 (29). Finally, Eriksson et al. have identified complement activation through the MBL pathway as a novel amplification mechanism that contributes to pathological thrombosis in critically ill COVID-19 patients (30).

Based on the multiple potential interactions between viral pathogens and complement, and principally on the above described associations we hypothesized that complement activation may be an important factor in the pathogenesis of SARS-CoV-2 infection. Accordingly, the aim of our work was to describe activities of the alternative and classical complement pathways, levels of their components, regulators and activation products in COVID-19 patients, and to search for associations between severity and outcome of COVID-19 and complement activation, dysregulation, and consumption.

## Patients and Methods

### Patient Selection

**Figure 1:**
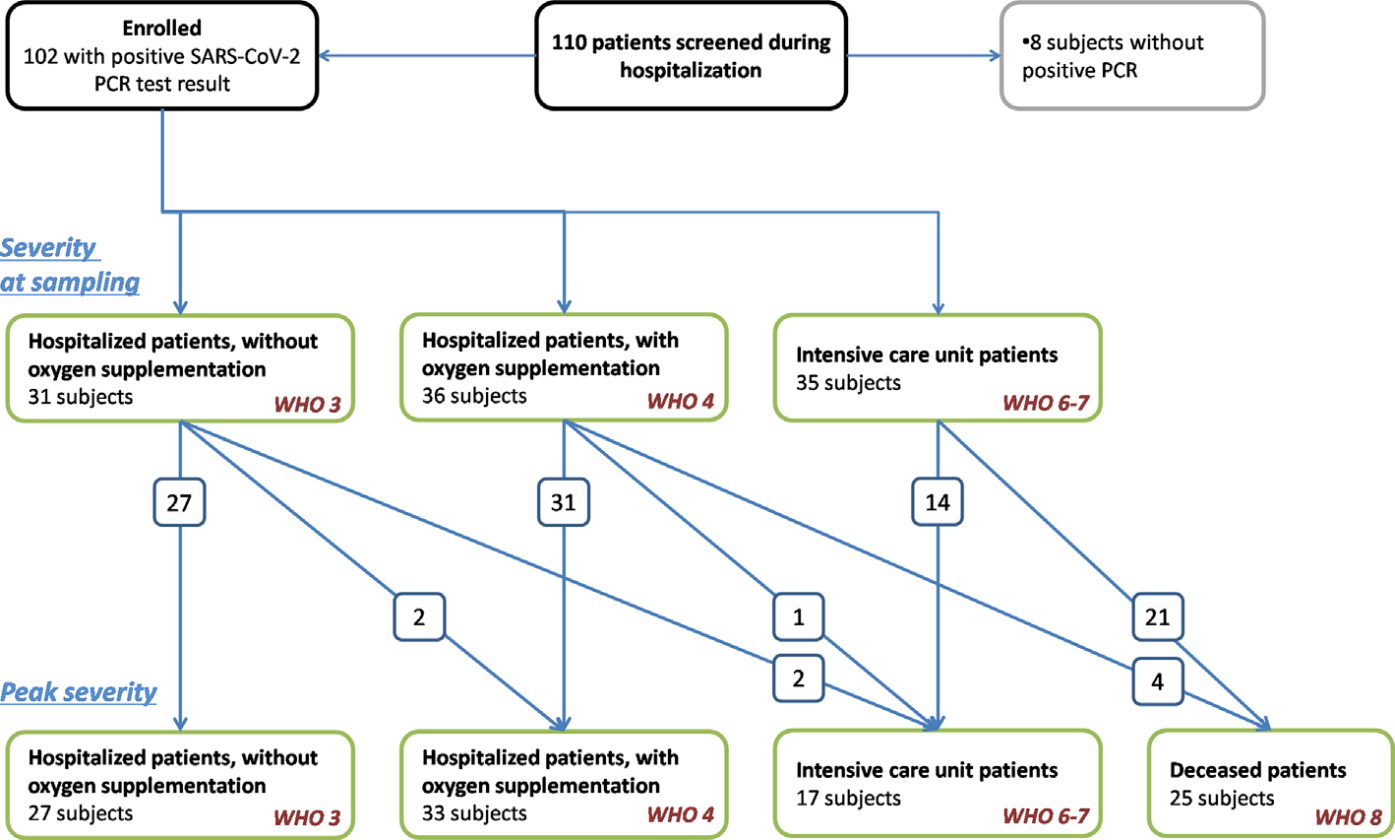
Study flow chart. Screening and enrollment of patients with SARS-CoV-2 infection. Only hospitalized patients with PCR-confirmed SARS-CoV-2 infection were enrolled into the study. Clinical and treatment data indicating severity of COVID-19 were extracted from electronic hospital records, and patients were stratified according to severity in two time-points: first, at the time point when sampling for complement analysis was done, and second, according to the worst clinical condition (or death). Arrows and numbers indicate the number of patients whose COVID-19 progressed, i.e. these patients progressed to an advanced severity group (or died) after sampling. Definitions for severity groups were based on WHO protocol (31). Note, that extra-corporeal membrane oxygenator treatment, non-invasive ventilation, or high-flow oxygen therapy was not used for patients in this study, therefore, there is no “WHO 5” severity group in this study. Patients, who were registered to donate convalescent plasma in a clinical trial (32), and who had evidence of past SARS-CoV-2 infection but did not require hospital treatment were sampled in convalescent phase formed the patient control group.

We enrolled an adult (age >18 years) cohort of SARS-CoV-2 infected hospitalized patients. One hundred and ten subjects, who received care for suspected COVID-19 in two tertiary hospitals in Budapest, were sampled and screened for inclusion into this study. Finally, to form groups as homogenous as possible, 102 hospitalized patients were included, as presented on the study flow chart (**Figure 1**). Inclusion criteria were: 1, COVID-19 disease confirmed by at least 1 positive SARS-CoV-2 RT-PCR test result from a nasopharyngeal swab specimen, 2, Available sample for complement analysis taken when the patient was hospitalized due to acute SARS-CoV-2 infection, 3, Accessible digital hospital record to extract clinical data. Patients, who were registered to donate convalescent plasma in a clinical trial (32), and who had evidence of past SARS-CoV-2 infection but did not require hospital treatment were sampled in convalescent phase, and formed the patient control group (CONTR). The study was approved by the Hungarian Ethical Review Agency (ETT-TUKEB; No. IV/4403-2/2020/EKU). Written informed consent was obtained from the patients, or from the closest relative available, if the patient was unable to give consent. The Declaration of Helsinki and its subsequent revisions were followed.

### Outcomes, Definitions

The primary outcome of this study was in-hospital, all-cause mortality, and the secondary outcome was severity of COVID-19. Assessment of disease severity was based on an eight-point ordinal scale as outlined by the WHO (31), using the following definitions: Hospitalized, but not critical patients were divided into two categories based on the requirement of supportive oxygen therapy [WHO category 3, “In-hospital patients without oxygen support” (HOSP); or WHO 4, “In-hospital patients with nasal oxygen support” (HOSP+O_2_)]. Critical patients included all patients who required intensive care unit (ICU) treatment for any organ support (WHO category 6 and 7, “critical”) and/or died (WHO 8, FATAL). Severity was first assessed when sampling was performed, and peak in-hospital severity was also registered (**Figure 1**).

### Samples

Blood samples (native- and EDTA-anticoagulated blood) were taken after fasting from the antecubital vein, or from a central venous catheter. Samples were transferred to the processing laboratory immediately after the sample was taken, cells and serum/plasma were separated by centrifugation, and aliquots were stored at −70°C until measurements.

### Laboratory Determinations

Fasting blood samples were used to measure standard clinical chemistry and inflammatory parameters and complete blood counts. Concentrations of C3 and C4 were measured by turbidimetry (Beckman Coulter, Brea, CA, USA). Total activity of alternative and lectin pathway was measured by a commercially available kit (Wieslab AP and LP ELISA KITs, KOMPL AP330 and KOMPL MBL320, Svar Life Science, Malmö, Sweden), according to the manufacturer’s instructions. Total classical pathway activity was measured by hemolytic titration test based on Mayer’s method (33). Radial immunodiffusion was performed to measure the antigenic concentrations of Factor I and Factor B, using specific polyclonal antibodies (34). Levels of Factor H and C1q were determined by homemade ELISA (34, 35). Complement activation markers such as sC5b-9 and C3a were detected by commercially available ELISA kits (MicroVue C3a-desArgEIA, A032; MicroVue sC5b-9 Plus EIA A029) in EDTA plasma sample.

### Statistical Analysis

Categorical data are reported as frequencies (%). As most of the variables showed skewed distributions, data are presented as medians and interquartile (IQ) ranges, and non-parametric statistical tests were used (Spearman rank correlation test, Mann–Whitney test) for two-, and Kruskal-Wallis test (with Dunn’s post test) for multiple independent groups). Uni- and multivariate Cox proportional hazards models were fitted to assess the effect of complement activation on primary outcome events. Survival times were measured from hospitalization until discharge of the patients from the hospital (including transmission to rehabilitation care) or death (all-cause, in-hospital mortality). The results of the Cox regression models are presented as hazard ratios (HR), the corresponding 95% confidence intervals (CIs), and the chi-squares and p values of likelihood ratio tests. Findings with p-values below 0.05 were considered statistically significant. Statistical calculations were performed with the GraphPad Prism 5 software (GraphPadSoftwares Inc., La Jolla, CA, USA) or by Statistica 13.5 (TIBCO Softwares Inc, Palo Alto, CA USA).

## Results

One hundred and two SARS-CoV-2 positive hospitalized patients were enrolled into this study (**Figure 1**). Twenty-six patients who were all outpatients when infected with SARS-CoV-2, and were sampled in convalescent phase, formed the patient control group [sampled at mean 53 (SD 13), min: 26, max: 74 days after onset of symptoms].

**Figure 2:**
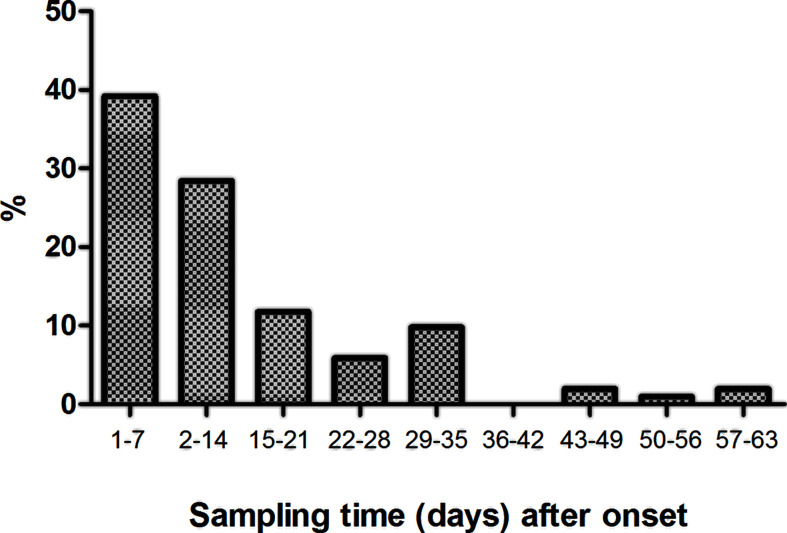
Histogram (fraction of patients) showing time delay (days) between onset of disease (first symptoms) and sampling.

In-hospital patients (n = 102) were divided into three severity subgroups (without or with nasal oxygen support, or necessitating intensive care unit treatment) when sampled for this study. Sampling was done when the patient was first hospitalized in Central Hospital of Southern Pest National Institute of Haematology and Infectious Diseases or in Semmelweis University, or when the patient was transferred from a different hospital to our centers. Therefore, the elapsed time between onset of symptoms and sampling varied between 1 day and 63 days, as shown on **Figure 2**. Typically, sampling was done on day 8 (median) after onset of symptoms [this is day 6 (median) after first PCR positivity]. Sixty-seven percent of patients were sampled on the first 2 weeks after onset, and the remaining one-third on weeks 3–9 (**Figure 2**). Extra-corporeal membrane oxygenator treatment, non-invasive ventilation, or high-flow oxygen therapy was not used for patients in this study, therefore, no patients fall into WHO category 5.

Severity of COVID-19 changed over time during the hospital stay (as presented on **Figure 1**), therefore, severity was assessed at a second time when “peak” was reached. Thirty-three in-hospital patients required nasal oxygen support during the hospital stay. During the observation period 25 patients died, 17 survivor patients were discharged from ICU. Baseline characteristics revealed significant differences across various severity categories in comorbidities, complications, and inflammatory markers of COVID-19 (**Table 1**). Severe COVID-19 disease was associated with frequent occurrence of diabetes mellitus and malignant diseases (p < 0.0001). Patients, who died later, had four comorbidities (median), whereas survivors had only two (**Table 1**). Increased numbers of in-hospital complications, such as pneumonia, respiratory failure, sepsis, thromboembolic events, and acute kidney injury, were more common among those who required ICU treatment or died (**Table 1**, p < 0.0001). Lymphopenia, increased neutrophil count, and gradually increasing IL-6, C-reactive protein (CRP), troponin, and ferritin levels were all present in patients with more severe forms of COVID-19 (**Table 1**).

**Table 1:** Basic characteristics of SARS-CoV-2 infected patients, comparison according to peak severity.

Variables	Total, n = 128	Outpatients, n = 26	Hospitalized, no oxygen support, n = 27	Hospitalized, with nasal oxygen support, n = 33	Critical, n = 17	Death n = 25	p-value*
Male sex, n (%)	71 (55.5)	15 (57.7)	17 (63.0)	20 (60.6)	8 (47.1)	11 (44.0)	0.429
Mean age ± SD	60.5 ± 16.5	44.5 ± 10.1	57.0 ± 16.1	68.3 ± 11.3	56.5 ± 15.2	75.3 ± 9.4	<0.0001
Total number of comorbidities (median, IQR)	2 (1–3)	0 (0–1)	2 (1–3)	2 (2–3)	2 (1–3)	4 (2–4)	0.016
Hypertension, n (%)	73 (57.0)	7 (26.9)	13 (48.2)	22 (66.7)	11 (64.7)	20 (80.0)	0.118
Chronic pulmonary disease, n (%)	22 (17.2)	0 (0)	3 (11.1)	6 (18.2)	4 (23.6)	9 (36.0)	0.165
Diabetes mellitus, n (%)	26 (20.3)	1 (3.8)	4 (14.8)	8 (24.2)	2 (11.8)	11 (44.0)	0.046
Chronic heart disease, n (%)	34 (26.6)	0 (0)	6 (22.2)	14 (42.4)	3 (17.7)	11 (44.0)	0.117
Malignant disease, n (%)	23 (18.0)	0 (0)	4 (14.8)	2 (6.1)	8 (47.1)	9 (36.0)	0.003
Other comorbidity, n (%)	89 (69.5)	1 (3.8)	26 (96.3)	28 (84.8)	11 (64.7)	23 (92.0)	0.885
Presenting symptoms							
Fever, n (%)	72 (56.7)	15 (57.7)	9 (33.3)	19 (57.6)	16 (94.1)	13 (52.0)	0.0033
Cough, n (%)	70 (54.7)	14 (53.8)	11 (40.7)	21 (63.6)	11 (64.7)	13 (52.0)	0.3480
Dyspnea, n (%)	57 (44.5)	3 (11.5)	7 (25.9)	16 (48.5)	12 (70.6)	19 (76.0)	<0.0001
Transfer to ICU, n (%)	38 (29.7)	0 (0)	0 (0)	0 (0)	17 (100)	21 (84.0)	<0.0001
Delay between first symptom and sampling, days (median, IQR)	9 (5–20)	–	12.5 (8–28)	8.5 (6–15)	10 (7–28)	6 (3–16)	0.136
Complications							
Pneumonia, n (%)	81 (63.3)	1 (3.8)	14 (51.9)	28 (84.8)	16 (94.1)	22 (88.0)	<0.0001
Respiratory failure necessitating mechanical ventilation, n (%)	30 (23.4)	0 (0)	0 (0)	0 (0)	10 (58.8)	20 (80.0)	<0.0001
Sepsis, n (%)	18 (14.1)	0 (0)	1 (3.7)	1 (3.0)	5 (29.4)	11 (44.0)	<0.0001
Thromboembolic complications, n (%)	14 (10.9)	0 (0)	3 (11.1)	0 (0)	7 (41.2)	4 (16.0)	<0.0001
Acute kidney injury, n (%)	13 (10.2)	0 (0)	0 (0)	2 (6.1)	2 (11.8)	9 (36.0)	0.002
Other complication, n (%)	36 (28.1)	0 (0)	7 (25.9)	10 (30.3)	6 (35.3)	13 (52.0)	0.0013
Death, n (%)	25 (19.5)	0 (0)	0 (0)	0 (0)	0 (0)	25 (100)	<0.0001
Total number of in-hospital complications (median, IQR)	1 (0–2)	0 (0–0)	0 (0–1)	1 (1–2)	2 (1–3)	2 (1–4)	<0.0001
Laboratory findings (median, IQR)							
Neutrophil granulocyte count (2–7.5 G/L)	4.1 (3.0–5.9)	3.9 (3.0–4.6)	3.8 (2.8–5.1)	3.8 (2.9–5.9)	5.0 (3.2–6.1)	6.1 (2.1–10.0)	0.0100
Lymphocyte count (1.5–4 G/L)	1.4 (0.9–1.9)	2.0 (1.8–2.4)	1.6 (1.0–2.2)	1.5 (1.0–1.9)	0.9 (0.8–1.3)	0.8 (0.5–1.1)	<0.0001
Interleukin 6 (2–4.4 pg/mL)	24.2 (7.1–67.9)	1.7 (1.1–2.5)	12.5 (5.6–24.5)	27.8 (9.5–63.8)	40.1 (14.3–51.3)	90.4 (34.6–267.3)	<0.0001
C-reactive protein (<10 mg/L)	29.4 (3.7–107.6)	1.3 (0.3–2.5)	11.6 (5.6–41.0)	36.8 (17.5–88.6)	111 (61.3–169.1)	149.1 (54.9–196.8)	<0.0001
D-dimers (<500 ng/mL)	1,130 (580–1,924)	207 (158–453)	1,460 (610–2,210)	851 (530–1,526)	1,658 (912–3,080)	1,430 (1,106–4,380)	0.009
Ferritin (15–300 ng/mL)	536 (261–1,146)	NA	320 (163–547)	379 (230–710)	1,321 (929–1,784)	702 (423–2,080)	<0.0001
Troponin (<34.0 ng/mL)	20.5 (5.0–51.0)	NA	13 (5–26)	13 (4–31)	15 (4–40)	51 (31–89)	0.0054

*p-values were obtained for nominal variables by the chi-square test, for continuous variables by the Kruskal-Wallis test. Results of outpatients are shown for reference only, this group was not included in the statistical analysis. NA, not applicable/not available.

Other comorbidities included: Acute myocardial infarction, stroke, chronic renal failure, chronic psychiatric diseases, dementia, epilepsy, sclerosis multiplex, Alzheimer’s disease, acute myeloid leukemia, chronic lymphoid leukemia, HIV infection.

Other complications included: pneumothorax, acute atrial fibrillation, urinary tract infection, C. difficile infection/enterocolitis, stroke, ileus, peripheral gangrene.

For laboratory markers reference ranges are indicated in brackets.

### Complement Profile in Relation to Severity of COVID-19

Results of complement parameters are shown in **Table 2** according to the patient’s actual severity stage when sampling was done (see above, and **Figure 2**). Levels of C3, C4, Factor B, and alternative pathway activity showed significant association with COVID-19 severity. Based on the results of post-tests C4 and Factor B levels were highest in hospitalized patients without ICU treatment. Lowest C3 concentration and AP activity were measured in patients in critical stage. The activity of lectin pathway showed no difference between severity groups. Complement activation products C3a and sC5b-9 showed increasing levels across severity groups with a highly significant association, and highest levels in critical patients. Complement parameters were associated with multiple markers of inflammation and coagulation, as shown in **Table 3**. Strong associations were noted between CRP and Factor B, C3a, sC5b-9; ferritin and C3a, sC5b-9; and haptoglobin and C3, C4, Factor B. D-dimer levels were inversely associated with concentrations of C3, C4, and activities of alternative and classical pathways, whereas positively associated with C3a. Among the complement factors, Factor B showed the highest level correlation with complement activation products (for C3a r = 0.286, p = 0.003; for sC5b-9 r = 0.328 and p = 0.0008).

**Table 2:** Complement profile of the SARS-CoV-2 infected patients, comparison according to severity when sampling.

Complement parameter*		Severity when sampling				
	Reference range	Outpatients, n = 26	Hospitalized, no oxygen support, n = 31	Hospitalized, with nasal oxygen support, n = 36	Critical, n = 35	p-value**
**Alternative pathway activity, %**	70–130	99 (88–104)	94 (82–103)	88 (67–108)	80 (54–96)^#^	0.0136
**Classical pathway activity, CH50/mL**	48–103	69 (58–79)	71 (67–86)	83 (63–93)	71 (45–85)	0.1082
**Lectin pathway activity, %**	25–125	53 (1–130)	55 (1–120)	113 (23–143)	46 (1–120)	0.069
**C3, g/L**	0.9–1.8	1.26 (1.10–1.42)	1.31 (1.11–1.49)	1.29 (1.10–1.45)	1.11 (0.73–1.37)	0.0443
**C4, g/L**	0.15–0.55	0.28 (0.20–0.33)	0.37 (0.26–0.56)^#^	0.36 (0.27–0.47)^#^	0.30 (0.18–0.51)	0.0108
**C1q, mg/L**	60–180	99 (90–121)	109 (82–128)	103 (87–134)	114 (85–145)	0.737
**Factor B, %**	70–130	90 (74–111)	110 (97–139)^##^	129 (90–154)^##^	118 (97–138)	0.0075
**Factor H, mg/L**	250–880	762 (485–856)	821 (484–1,056)	739 (535–1,051)	640 (337–865)	0.167
**Factor I, %**	70–130	95 (72–111)	102 (84–119)	109 (91–118)	95 (80–119)	0.483
**C3a, ng/mL**	70–270	122 (95–171)	235 (117–292)^#^	210 (140–310)^##^	398 (230–574)^###^	<0.0001
**sC5b-9, ng/mL**	110–252	183 (143–254)	245 (168–374)	307 (220–395)^##^	365 (251–556)^###^	0.0001

* All data presented as median (IQR). **p-value based on Kruskal-Wallis test; hashtags indicate results of Dunn’s post test (^#^p < 0.05, ^##^p < 0.01, and ^###^p < 0.001), when compared to outpatients.

**Table 3:** Correlation between complement parameters and markers of inflammation and fibrinolysis.

	Interleukin 6 (pg/mL)	C-reactive protein (mg/L)	Haptoglobin (g/L)	Ferritin (ng/mL)	D-dimers (ng/mL)
**Alternative pathway activity, %**	−0.172 (0.087)*	0.023 (0.817)	**0.215 (0.031)**	−0.068 (0.507)	**−0.287 (0.011)**
**Classical pathway activity, CH50/mL**	−0.074 (0.462)	0.118 (0.237)	**0.345 (0.0003)**	−0.006 (0.953)	−0.209 (0.066)
**Lectin pathway activity, %**	0.094 (0.393)	0.016 (0.882)	0.152 (0.150)	0.069 (0.545)	**−0.224 (0.049)**
**C3, g/L**	−0.114 (0.256)	0.069 (0.492)	**0.508 (<0.0001)**	−0.041 (0.693)	**−0.234 (0.039)**
**C4, g/L**	0.053 (0.599)	0.191 (0.056)	**0.285 (0.004)**	0.062 (0.548)	**−0.282 (0.012)**
**C1q, mg/L**	0.129 (0.202)	0.211 (0.036)	0.004 (0.966)	0.119 (0.252)	−0.054 (0.639)
**Factor B, %**	0.081 (0.421)	**0.423 (<0.0001)**	**0.478 (<0.0001)**	0.061 (0.556)	−0.038 (0.740)
**Factor H, mg/L**	0.014 (0.889)	**0.208 (0.037)**	**0.424 (<0.0001)**	0.123 (0.231)	−0.178 (0.118)
**Factor I, %**	0.025 (0.802)	0.152 (0.128)	**0.391 (0.0002)**	0.016 (0.876)	−0.041 (0.720)
**C3a, ng/mL**	**0.313 (0.001)**	**0.623 (<0.0001)**	0.154 (0.126)	**0.387 (0.0001)**	**0.227 (0.048)**
**sC5b-9, ng/mL**	0.136 (0.182)	**0.409 (<0.0001)**	**0.223 (0.025)**	**0.227 (0.027)**	0.114 (0.326)

*Spearman correlation coefficients and p-values are presented. Bold indicates significant associations (p < 0.05).

**Figure 3:**
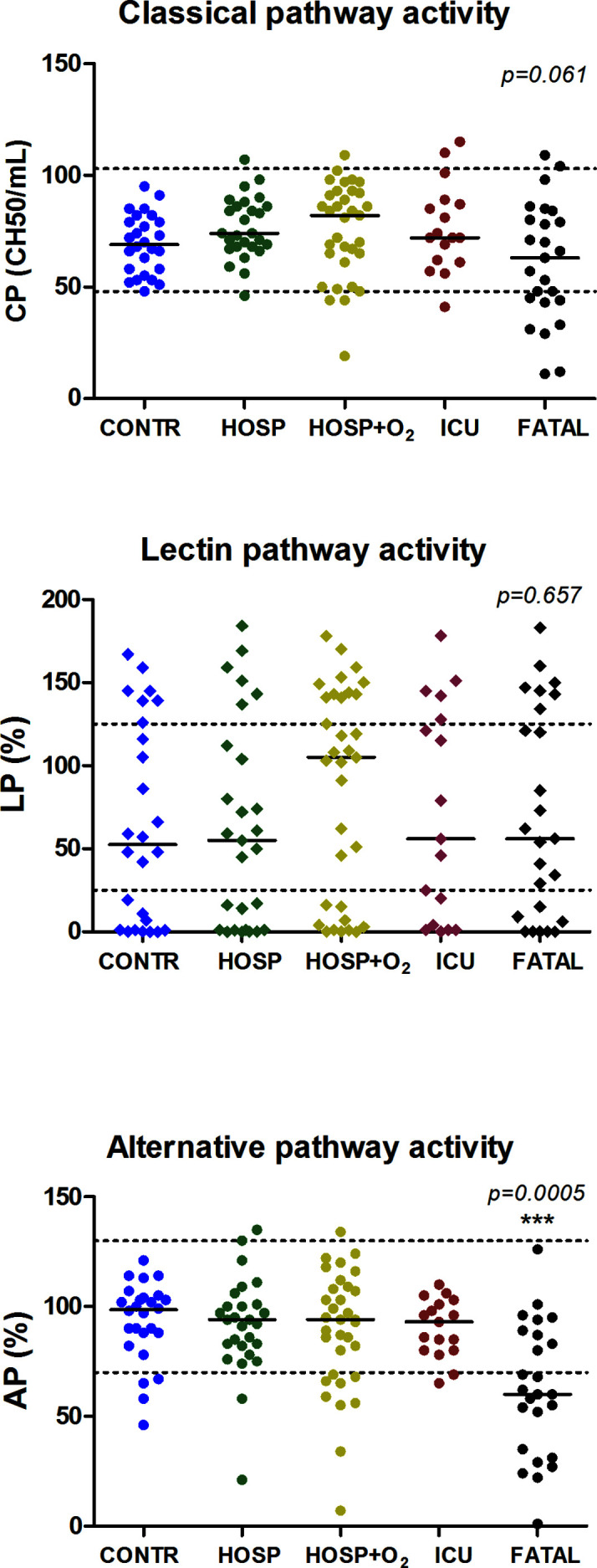
Association between complement functional activity and peak severity of SARS-CoV-2 infection. The horizontal solid lines indicate group median, horizontal dashed lines indicate reference range limits, p-values were obtained by Kruskal-Wallis test; asterisks indicate results of Dunn’s post test (***p < 0.001), when compared to control.

**Figure 4:**
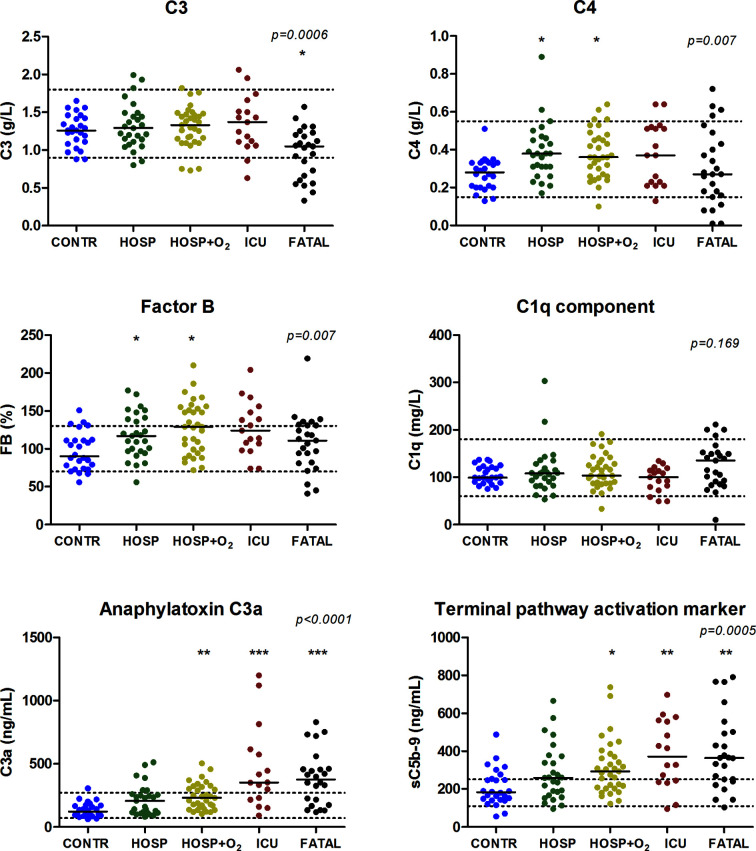
Association between complement parameters and peak severity of SARS-CoV-2 infection. The horizontal solid lines indicate group median, horizontal dashed lines indicate reference range limits, p-values were obtained by Kruskal-Wallis test; asterisks indicate results of Dunn’s post test (*p < 0.05, **p < 0.01, and ***p < 0.001), when compared to control.

**Figures 3** and **4** show individual results of complement profiles in patient groups according to peak severity. The most remarkable difference in complement parameters is in the group of critical patients: declining C3, C4, Factor B levels, alternative and classical pathway activities are all characteristic for non-survivor patients, when compared to survivors. Remarkably, deceased patients had the highest level of C1q, although C1q levels were not increased in the less severe groups of patients. Among complement parameters measured, complement activation products (anaphylatoxin C3a and terminal pathway activation marker sC5b-9) showed the strongest associations with severity of COVID-19. Significantly elevated C3a and sC5b-9 levels were observed in groups requiring oxygen support, ICU-treatment, and in the group of deceased patients, when compared to controls (**Figures 3** and **4**).

**Figure 5:**
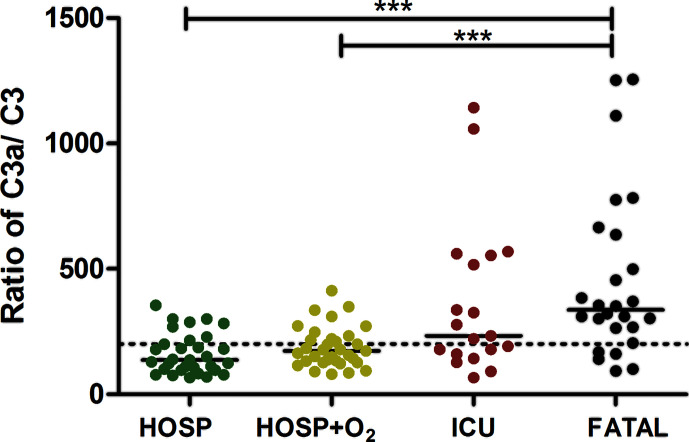
Association of complement C3 overactivation and consumption, and peak severity of COVID-19. Ratios of complement anaphylatoxin C3a/C3 (Kruskal-Wallis test p < 0.0001, ***Dunn’s post test p < 0.001) are presented in patient groups stratified according to peak severity. Cut-point (200) used in the survival analysis is indicated by horizontal, dashed line.

**Figure 6:**
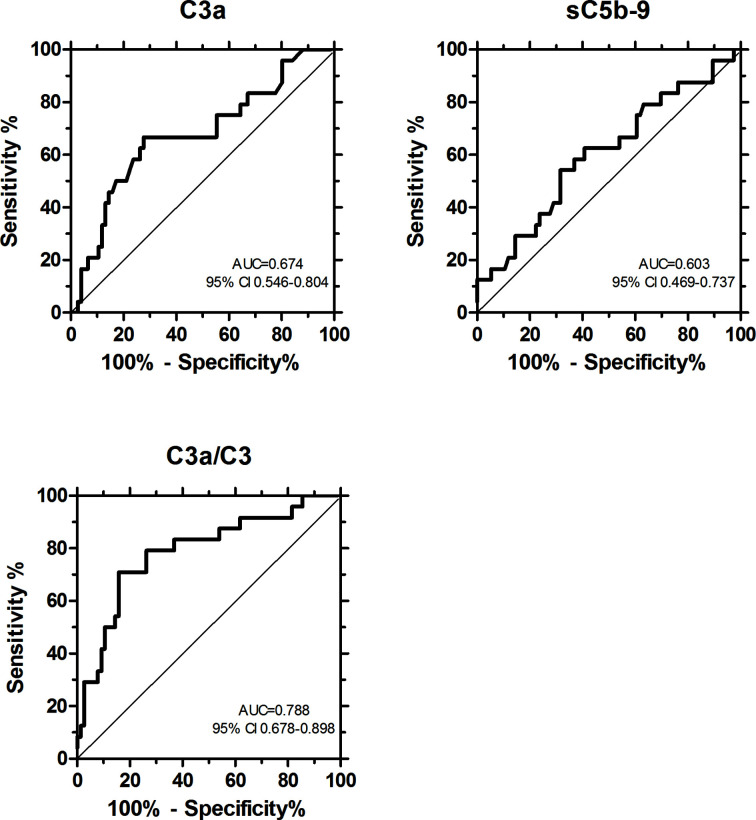
Receiver-operator characteristics (ROC) analysis to evaluate the relationship between complement markers and mortality, and to obtain optimum cut-points differentiating survivor and non-survivor patients. ROC curves with C-statistics and 95% confidence interval are shown.

In order to further analyze the role of overactivation and consumption of complement in COVID-19, the ratio of C3a/C3 was calculated. **Figure 5** shows individual C3a/C3 in various severity groups of hospitalized COVID-19 patients, and showed strong association with severity (Kruskal-Wallis ANOVA p < 0.001). Significant elevation of C3a/C3 ratio was characteristic for non-survivor critical patients, when compared to non-critical patients (**Figure 5**). To analyze if C3a, sC5b-9, and C3a/C3 ratio are appropriate markers to differentiate between survivor *vs.* non-survivor patients, receiver-operator characteristics analysis was done (**Figure 6**). C-statistics were significantly higher than 0.5 for C3a (0.674) and C3a/C3 (0.788) while sC5b-9 marker was not significant in this analysis. Based on the ROC analysis, cut points of 324 ng/mL (for C3a) and 200 (for C3a/C3) were selected for further multivariable survival analysis.

### Prediction of In-Hospital Mortality by Complement Overactivation and Consumption

**Figure 7:**
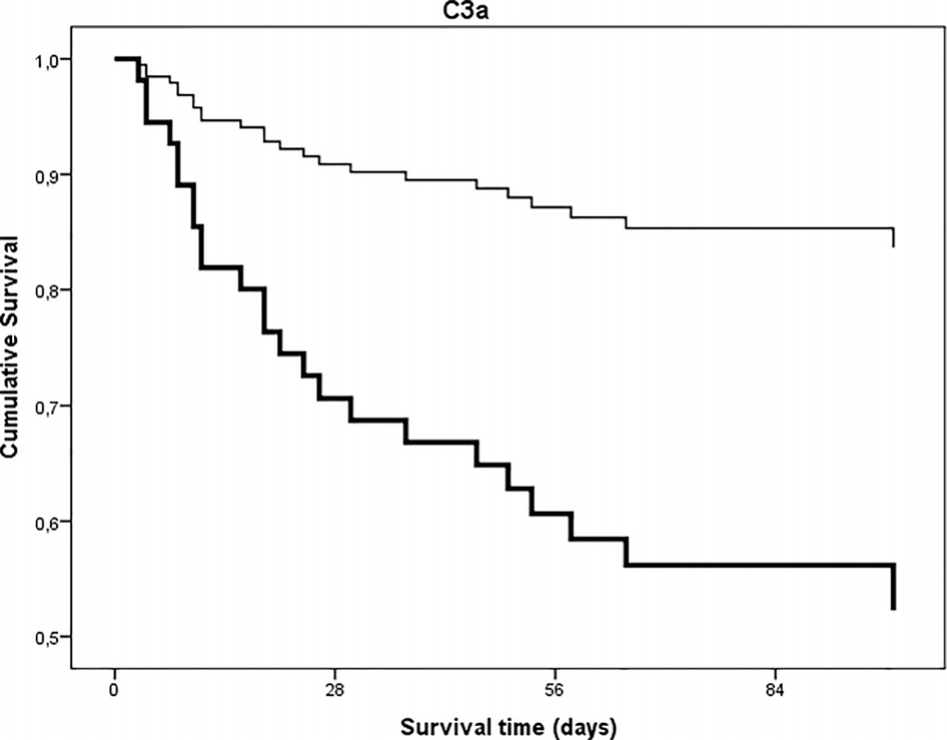
Univariate time to in-hospital, all-cause mortality Cox proportional-hazards regression analysis in patients with COVID-19 as stratified by level of anaphylatoxin C3a. Patients were stratified according to C3a levels (cut point 324 ng/mL p = 0.002).

**Figure 8:**
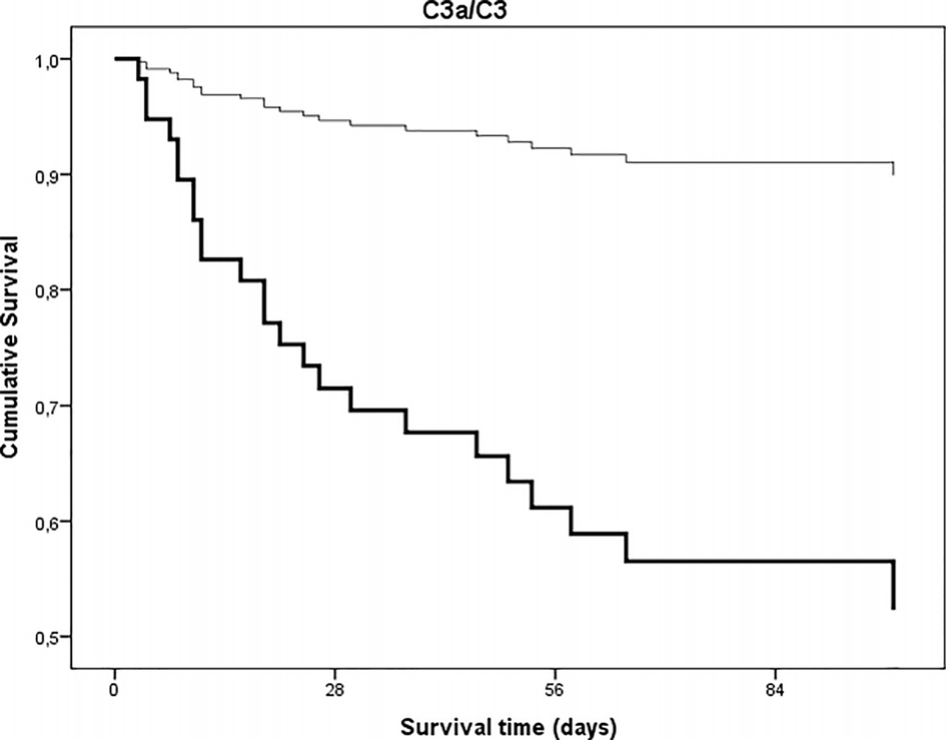
Univariate time to in-hospital, all-cause mortality Cox proportional-hazards regression analysis in patients with COVID-19 as stratified by extent of complement C3 overactivation and consumption. Patients were stratified according to ratios indicating complement C3 overactivation and consumption (C3a/C3 ratio, cut point 200, p < 0.0001).

Twenty-five (24.5%) of the 102 hospitalized patients of this study died during hospital stay. First, we analyzed whether anaphylatoxin C3a, or the ratio of C3a/C3 were associated with mortality of hospitalized COVID-19 patients. The median (interquartile range) of C3a, and C3a/C3 ratio among survivors was 236 ng/mL (140–337) and 179 (123–270), *versus* 375ng/mL (195–459, p = 0.009) and 337 (250–644, p < 0.0001, Mann-Whitney test) among non-survivors, respectively. Next, the groups of patients with high *versus* low levels of C3a, and complement overactivation and consumption (C3a/C3, both as categorized variables) were compared regarding in-hospital mortality. In the group of low (<324 ng/mL) C3a or low (<200) C3a/C3 mortality was 13.0 and 8.2%, respectively, whereas for high C3a or high overactivation and consumption groups mortality was 43.6 and 40.8%, respectively. Further, Cox proportional-hazard survival models were generated to analyze the effects of complement overactivation and consumption on in-hospital mortality. Univariate models (**Table 4**) showed significantly higher hazard ratios for those who have high levels of C3a, or high complement overactivation and consumption (C3a/C3), when compared to those with low levels. In particular, having C3a/C3 above 200 translates into 6.1 times (2.1–17.8, 95% CI) higher risk of death, when compared to those with <200 ratio. **Figures 7** and **8** show univariate Cox survival curves of COVID-19 patients with or without complement overactivation and consumption. Importantly, in separate multivariable models, after adjustment for total number of comorbidities, or total number of complications, or for CRP, the prototype inflammatory marker, high level of C3 overactivation and consumption remained significant predictors of in-hospital mortality in COVID-19 patients (**Table 4**). Due to the variance of delay time between onset of symptoms and sampling (**Figure 2**), the Cox models were adjusted for this confounder, too. The association between complement overactivation and consumption (C3a/C3) and mortality remained significant in these adjusted multivariate models (**Table 4**). Finally, as no standard observation time was used in this study, logistic regression analysis with dead or alive, as binary outcome, was also done to describe the associations between complement overactivation. Patients with C3a/C3 ratio above 200 had 12.58-times higher chance for fatal outcome (95% CI 3.391–40.47), when compared to patients with <200. If this association was adjusted for age, total number of comorbidities, or total number of complications (in separate models), the association remained significant (data not shown).

**Table 4:** Results of univariate and multivariable Cox proportional-hazards regression models analyzing effects of complement overactivation in-hospital mortality.

Model	HR	95% CI	Chi-square	p-value
	C3a*			
Univariate	3.636	1.556–8.499	9.606	0.002
Adjusted for age	3.093	1.322–7.235	7.315	0.007
Adjusted for total number of comorbidities	2.978	1.237–7.168	6.303	0.012
Adjusted for total number of complications	2.150	0.813–5.688	2.472	0.116
Adjusted for C-reactive protein	2.346	0.898–6.127	3.155	0.076
Adjusted for delay between onset of symptoms and sampling	2.895	1.151–7.281	5.421	0.020
	C3a/C3 ratio*			
Univariate	6.1	2.08–17.87	15.01	<0.0001
Adjusted for age	3.90	1.31–11.58	7.71	0.005
Adjusted for total number of comorbidities	4.98	1.66–14.89	10.78	0.001
Adjusted for total number of complications	4.06	1.29–12.85	6.96	0.008
Adjusted for C-reactive protein	4.430	1.434–13.684	8.298	0.004
Adjusted for delay between onset of symptoms and sampling	4.909	1.638–14.709	10.325	0.001

*C3a level and C3a/C3 ratio was analyzed as categorical variable, differentiating patients with high or low level of complement activation (cut points were 324 ng/mL for C3a; and 200 for C3a/C3).

HR, Hazard ratio; CI, confidence interval.

## Discussion

Our study provides the first observational proof that biomarkers of complement overactivation and consumption are useful tools for the prediction of in-hospital mortality of COVID-19 patients. We observed a continuous increase of complement activation markers C3a and sC5b-9 across WHO categories of COVID-19 severity (**Figure 4** and **Table 2**), with highest levels in patients presenting with critical illness. C3 concentrations were remarkably decreased in the group of non-survivors (**Figure 4**), with significantly elevated C3a/C3 ratios, reflecting overactivation and consumption (**Figure 5**). This difference translated to significant prediction of mortality in Cox proportional-hazards survival models, with hazard ratios of 6.1 (2.1–17.8, 95% CI) for those having increased ratio of C3a/C3. The model remained significant after adjustment for important covariates of critical illness, including comorbidities, in-hospital complications, or CRP.

The demonstration of complement C3 overactivation and consumption, and its strong association with COVID-19 severity and outcome, is intriguing and confirms recent observations. First, the RCI-COVID-19 group demonstrated activation of complement with elevated C3a, sC5b-9, and C3c levels in severe COVID-19 (27). High levels of complement activation markers were associated with mortality and thromboembolic complications, however, prediction of mortality was not formally evaluated in survival models. Recently, the activation through the MBL pathway and pathological thrombosis in critically ill COVID-19 patients was described (30), highlighting the critical role of complement activation in important complications of SARS-CoV2 infection. In addition, Fang et al. (19) reported low serum C3 levels in association with COVID-19 related mortality. Specifically, their study identified approximately seven times higher risk of death, per 1 g/L decrease in serum C3 level. In the same study complement activation has not been analyzed, hence whether decreased C3 was due to overactivation and consumption, or protein loss/decreased production, remained elusive. Of note, complement activation was shown to predict COVID-19 progression in chronic hemodialysis patients (36), and C5a was considered as an earlier marker, than C3a. Furthermore, we also observed decreased C4 levels in the group of non-survivors, indicating a potential involvement of the lectin or classical pathways behind complement activation and consumption. It is of note, that levels of C1q antigen [a protein produced mainly by monocytes and macrophages (37)] were the highest in deceased COVID-19 patients, suggesting the potential role macrophage overactivation in the fatal outcome of the disease (38). These observations require detailed molecular analysis in future studies.

Importantly, our results provide observational support to the milestone paper of Yu et al. published in the journal Blood. The group at Johns Hopkins University reported that SARS-CoV-2 spike proteins bind heparan sulfate on cell surface and overactivate mainly the alternative pathway by interfering with its main soluble regulator, Factor H. Moreover, ACH145951, a small molecule Factor D inhibitor, blocked SARS-CoV-2 spike protein induced AP activation (39). Our clinical observations on the strong prediction of in-hospital mortality by the complement C3 overactivation and consumption provide independent observational support to the conclusion of the authors, who suggested that SARS-CoV-2 spike protein induced AP activation may have profound implications in the multiorgan dysfunction, coagulopathy, and endothelial injury, all characteristic of COVID-19.

Finally, in their elegant retrospective study Ramlall et al. (40) reported that history of age-related macular degeneration (AMD)—a condition known to be linked to complement dysregulation—is a risk factor for COVID-19 morbidity and mortality. In addition, with a candidate-driven genetic association study authors identified multiple putative risk loci in genes of complement regulators and components that were associated with clinical outcome of SARS-CoV-2 infection (40). Authors concluded that history of AMD predisposes patients to poor clinical outcome following SARS-CoV-2 infection, and variants in critical regulators of complement are associated with this inferior outcome. By showing the strong association between complement dysregulation (overactivation and consumption, as expressed by the C3a/C3 ratio, **Figure 5**) and severity and mortality of COVID-19, our current results corroborate the findings of Ramlall et al. Lastly, the C3 S/F polymorphism has been reported as a potential confounder of COVID-19 related mortality (41). C3 S/F polymorphism is functionally active (42), the F allele, being reported as a confounder of increased mortality, has also been associated with increased tendency to activate the alternative pathway. Therefore, inherited factors determining the activity and regulation of complement activation might indeed play important roles in the pathogenesis of COVID-19.

Inflammatory markers were closely related to COVID-19 severity (**Table 1**), which is an observation already reported at the beginning of the pandemic (43). Among the complement factors, Factor B, the enzymatic component of alternative pathway C3-convertase, was most closely related to inflammatory markers (**Table 3**) and showed the highest elevation across severity categories. Complement activation markers C3a and sC5b-9 showed significant, constant increase across severity groups of COVID-19 patients (**Table 2**, **Figure 4**). It is tempting to speculate that the particular tendency for complement overactivation and the “cytokine storm” after SARS-CoV-2 infection is based on the strong induction of hepatic acute phase reaction (including for example Factor B). Due to the high level and availability of alternative pathway components C3 and Factor B, complement activation with overproduction of inflammatory and cell damaging activation products is constant and increasing across all severity categories of COVID-19, and it is most probably the exhausting regulatory capacity that makes individuals prone to complement overactivation and consumption, and finally to death. Results of genetic association studies seem to support this assumption (40).

In COVID-19 viral pneumonia, acute respiratory distress syndrome and respiratory failure are frequent, life-threatening complications often linked to thromboinflammation and vascular damage. Analyzing pulmonary biopsy and autopsy samples of SARS-CoV-2 infected patients with immunochemistry, Magro and coworkers were the first to describe septal capillary injury accompanied by extensive deposits of MASP-2, C4d, and terminal complement complex (44). Authors concluded that COVID-19 associated pauci-immune, complement mediated lung injury is distinct from the typical ARDS. Supporting the contribution of systemic complement activation to the development of COVID-19 associated respiratory failure are the recent observations of Holter et al. (26). Authors observed that increased admission C4d and sC5b-9 levels were associated with the development of respiratory failure and higher need for oxygen therapy, and that complement activation correlated to anti-viral antibody and ferritin levels, but not to viral load. Our observations are in line with these results, since there were significant increases in C3a and sC5b-9 levels in correlation with COVID-19 severity, with highest levels in critical patients often also having respiratory failure (**Figure 4** and **Table 2**). Furthermore, complement factor and activation product levels were associated with CRP and ferritin concentrations (**Table 3**), reflecting the relationship between the extent of inflammation and complement dysregulation.

An important novel observation of the current study is the association of mortality in COVID-19 patients with markers of complement overactivation and consumption, independent of comorbidities and in-hospital complications. A prospective cohort study from New York City identified higher age, decreased oxygen saturation at presentation, comorbidities (chronic heart and renal failure and malignant disease), and increased C-reactive protein, D-dimer, procalcitonin, and troponin levels as strong predictors of in-hospital mortality (45). Since in-hospital complications, including development of respiratory failure, sepsis, thromboembolic complications, and renal failure, are strong determinants of progression to critical illness and intensive care unit treatment, we adjusted the Cox survival models for the total number of in-hospital complications. Prediction of mortality by markers of complement overactivation and consumption remained still significant after adjustment for in-hospital complications (**Table 4**). Adjustment (in separate models) for age and C-reactive protein or delay between disease onset and sampling, did not change the results, either (**Table 4**).

Current evidences support that SARS-CoV-2 infection leads to immune dysfunction, widespread endothelial injury, complement associated coagulopathy, and systemic microangiopathy (29). Our data reinforce the importance of clinical trials with complement inhibitors to limit complement mediated tissue damage and to decrease mortality in COVID-19. Multiple available inhibitors of complement cascade, including anti-C5 monoclonals eculizumab (17, 18, 46) and ravulizumab (19, 47), narsoplimab, a monoclonal antibody against MASP-2 (48), the compstatin-based complement C3 inhibitor AMY-101 (49), C1-inhibitor (50), and the anaphylatoxin C5a blocking antibody IFX-1 (vilobelimab) (51) are currently being evaluated for COVID-19. Preliminary observations on safety and efficacy results are promising (20), but it needs more time to make firm conclusions on the utility of such drugs for COVID-19.

This study was limited by its relatively small size enrolling 102 in-hospital patients (25 non-survivors) with confirmed COVID-19 in two tertiary care centers. However, the severity groups were nearly equally sized and follow-up was complete for all of the patients, allowing for reliable statistical analysis. *Post-hoc* power analysis yield P > 0.8 for both C3a and sC5b-9, when comparing the 25 non-survivors and the 27 in-hospital patients without need for oxygen support (**Figure 4**). The low number of non-survivors in the study allowed adjustment for only one confounder, but since our study collected all of the relevant clinical data about comorbidities and in-hospital complications, we were able to adjust for the most important confounders (**Table 4**). One of the strengths of our study was the detailed characterization of large panel complement profile by methods regularly evaluated in external proficiency testing (25). In addition, our strategy to measure complement activity and factor levels together with activation markers allowed us to identify the clinical relevance of complement overactivation together with signs of consumption, a phenomenon indicating the presence of complement dysregulation. Results on the relationship between activity of lectin pathway and disease severity are heavily influenced by genetic factors strongly regulating mannose-binding lectin levels, but due to space limitations, the results of detailed analysis will be presented separately (manuscript in preparation).

### Conclusion

Patients with SARS-CoV-2 infection are more likely to die, when the diseases is accompanied by overactivation and consumption of C3. These results may provide observational evidence and further support to studies on complement inhibitory drugs for the treatment of COVID-19.

## Data Availability Statement

The raw data supporting the conclusions of this article will be made available by the corresponding author, without undue reservation.

## Ethics Statement

The studies involving human participants were reviewed and approved by Hungarian Ethical Review Agency (ETT-TUKEB). Written informed consent to participate in this study was provided by the participants’ legal guardian/next of kin.

## Author Contributions

BM, ZF, DC, LH, EK, LC, and PK designed and performed laboratory determinations, interpreted data, and drafted the manuscript. GS, ZZP, and ZP conceptualized research, collected and analyzed clinical information and laboratory data, conducted statistical analysis, interpreted data, and wrote the manuscript. MR, VM, ZI, JG, LG, PR, BS, BL, JS, IB, TM, and IV-N conceptualization, collected and analyzed clinical information, interpreted and supervised data, and drafted the manuscript. All authors contributed to the article and approved the submitted version.

## Funding

The research was financed by the Higher Education Institutional Excellence Programme of the Ministry of Human Capacities in Hungary, within the framework of the molecular biology thematic program of the Semmelweis University, by the National Office for Innovation and Research [KH130355, and “Befektetés a jövőbe” (2020-1-1-6-JÖVŐ-2021-00013) to ZP]. The study was performed in frame of the Premium Postdoctoral Fellowship Program of the Hungarian Academy of Sciences (PPD2018-016/2018 to DC). ZP and LH are supported by funds of the EU MSCA project CORVOS 860044.

## Conflict of Interest

The authors declare that the research was conducted in the absence of any commercial or financial relationships that could be construed as a potential conflict of interest.
